# Stochastic processes in gravitropism

**DOI:** 10.3389/fpls.2014.00674

**Published:** 2014-11-26

**Authors:** Yasmine Meroz, Renaud Bastien

**Affiliations:** Applied Mathematics, School of Engineering and Applied Science, Harvard UniversityCambridge, MA, USA

**Keywords:** stochasticity, gravitropism, signal amplification, gradual response, stochastic resonance, noise, cytoskeleton

## Abstract

In this short review we focus on the role of noise in gravitropism of plants – the reorientation of plants according to the direction of gravity. We briefly introduce the conventional picture of static gravisensing in cells specialized in sensing. This model hinges on the sedimentation of statoliths (high in density and mass relative to other organelles) to the lowest part of the sensing cell. We then present experimental observations that cannot currently be understood within this framework. Lastly we introduce some current alternative models and directions that attempt to incorporate and interpret these experimental observations, including: (i) *dynamic sensing*, where gravisensing is suggested to be enhanced by stochastic events due to thermal and mechanical noise. These events both effectively lower the threshold of response, and lead to small-distance sedimentation, allowing amplification, and integration of the signal. (ii) The role of the cytoskeleton in signal-to-noise modulation and (iii) in signal transduction. In closing, we discuss directions that seem to either not have been explored, or that are still poorly understood.

## 1. Introduction

Tropism is the reorientation of a plant organ according to the direction of an external stimulus, one example of which is gravitropism (reorientation relative to the direction of gravity). While the first scientific investigations took place roughly 300 years ago (Dodart, 1703; Astruc, 1709), fundamental aspects of underlying mechanisms still remain elusive. Generally, the main steps of tropisms include the sensing of a stimulus, transduction of a signal, and lastly the response of the organ. Any sensory system is confronted with three main challenges: (i) sensitivity to weak stimuli, (ii) robustness to noise (be it internal or external), and (iii) a gradual response. Sensorimotor systems in other living organisms are known to be affected by noise at any one of these stages, and a large body of work investigates these phenomena (Mortensen and Suhl, 1991; Douglass et al., 1993a; Patel and Kosko, 2005; Faisal et al., 2008; Li et al., 2012). Only in the recent past has the role of noise been known to play a crucial role in tropisms. We note that all types of tropism rely on exquisitely sensitive and robust sensors. In light of this, the principal aim of this review is to establish the role of noise in gravitropism. We shall present the conventional model of gravitopism, and then point out a number of experimental observations that do not comply with it, while identifying various sources of noise at different stages of signal perception and transduction. Finally we shall indicate current alternative models and directions that attempt to incorporate these experimental observations, focusing on the role of noise.

## 2. Gravitropism

Gravitropism occurs both in roots and shoots. Roots manifest positive gravitropism, reorienting themselves toward the direction of gravity, while shoots manifest negative gravitropism, reorienting in the opposite direction (Sack, 1991). Cells, called statocytes, have been identified as be specialized in sensing gravity. In roots these are located in the root cap, and in shoots they form a cylindrical sheath surrounding the stem along the shoot (Morita and Tasaka, 2004). Recognizing that gravity acts on mass, the perceiving elements of these sensory cells have generally been identified as organelles with high mass and density (relative to other organelles). These organelles are termed *statoliths*. Starch-filled organelles called *amyloplasts* have been determined to play this role, though not exclusively as shall be seen later (Blancaflor and Masson, 2003; Perbal and Driss-Ecole, 2003; Morita and Tasaka, 2004; Moulia and Fournier, 2009). Upon displacement of the organ from its vertical orientation, the statoliths sediment to the new low point of the sensing cell. The cell is then able to recognize this change in orientation and transmits an associated signal. However, we shall see in the next Section that this picture does not account for a number of experimental observations.

Before proceeding it is interesting to note that stochastic processes dictate the bounds of both the sensitivity of gravity sensors (through thermal noise) and their selectivity (through the activation energy) (Björkman, 1988; Hasenstein, 2009). The sedimentation of statoliths is a mechanical process, and as such is affected by both mechanical and thermal noise (Björkman, 1988, 1992). Both gravity and thermal energy cause the gravity sensor to move, but thermal energy depends on temperature alone, and not on mass. Therefore, thermal energy acts as background noise that the gravity sensor must overcome, constituting a lower bound on sensitivity (Perbal et al., 2002). As for selectivity, the sensor should be selectively activated through a reaction by the stimulus rather than thermal motion. Higher selectivity can be obtained by raising the activation energy of the reaction high enough so that it is rarely stimulated spontaneously, but this also translates to a higher threshold stimulus, meaning that there is a trade-off between the sensor's sensitivity and its selectivity (Björkman, 1988, 1992).

## 3. Caveats of the classic model of amyloplast sedimentation: experimental observations

We present a few experimental observations that do not agree with the classical model of static sedimentation of amyloplasts. firstly, presentation time (the shortest stimulus needed for a response) can be as short as 7 s (Larsen, 1969). the average amyloplast sedimentation rate is estimated to be of the order of 0.154 μ*m*/min (Perbal, 2009). given that the width of a statocyte cell is of the order of 10 μ*m*, it should take the amyloplast several minutes to sediment to the bottom – a couple of orders magnitude longer than the mentioned presentation time.

Gravitropic response has been found to be highly sensitive; experiments carried out in microgravity or clinostatting, have revealed a threshold of 10^−4^ *g* (Shen-Miller et al., 1968; Merkis et al., 1985). Such high sensitivity requires large responding masses such as amyloplasts, and signal averaging. Indeed oat coleoptiles respond equally to 1 s stimuli administered every 5 s, and to 0.5 s stimuli administered every 1 s (Raven and Rubery, 1982), also suggesting signal averaging or integration.

Mutants with 60% of the starch of the wild type have been found to exhibit almost the same degree of sensitivity as the wild type (Kiss et al., 1996). In starchless mutants statoliths are lighter and therefore do not sediment. However, these mutants are still able to sense gravity, though with increased variability in the shoots orientation (Caspar and Pickard, 1989; Kiss et al., 1989; Vitha et al., 2000). Starchless plants therefore seem to rely on smaller masses, leading to an increased relative noise, as exhibited by the increased variability. Indeed apart from amyloplasts, prominent in higher plants, other statoliths have been identified: Chara relies on BaSO4-enriched vesicles (Hemmersbach et al., 1999), and fungi such as Phycomyces rely on protein crystals (Schimek et al., 1999). These statoliths have a much lower mass compared to amyloplasts, inconsistent with the requirement of large responding masses, and making them considerably weaker sensors.

Ma and Hasenstein (2007) investigated the possible dependency of gravisensing on thermal or mechanical noise, by studying the effect of temperature and external mechanical oscillations on the rate of gravitropic curvature on flax. They found that vertical oscillations prior to reorientation enhance gravisensing. The most effective enhancement occurred for oscillations of around 5 Hz and 0.5 mm amplitude (10 or 20 Hz did not promote curvature), reducing the time to reach half-maximal curvature by 18 min compared to non-oscillated roots (almost half the time). Similarly to mechanostimulation, temperature was also found to enhance gravitropic response. For vibration and temperature studies, the presentation times decreased almost 6-fold. Together with other work where mechanoperception was found to be enhanced by temperature (Douglass et al., 1993a,b), this may suggest that thermal noise plays a role in the process. The extent of this role needs to be better quantified since temperature also affects other biological processes, some of which, as shall be seen later, affect sensitivity.

The word statolith is derived form the Greek, meaning “stationary stone” – however saltatory movement of statoliths have been recorded (Morita, 2010): for example amyloplasts in corn coleoptiles (Sack et al., 1984), in maize root columella cells (Sack et al., 1986), and in the stem endodermis (Saito et al., 2005). In some cases the origin of the saltatory movements has been identified: actin-based lifting of statoliths in Chara rhizoids (Braun et al., 2002), in Arabidopsis endodermal cells amyloplasts perform F-actin-dependent saltatory movements (Saito et al., 2005). Indeed some of these saltations are non-Brownian movements constituting a simple type of cytoplasmic streaming (Sack and Leopold, 1985).

Recent work has identified the actin cytoskeleton as a crucial component in plant gravitropism (Blancaflor, 2002, 2013), both at the level of signal perception and transduction. Disrupting F-actin enhances sensitivity and causes an over-response in curvature for roots (Hou et al., 2003, 2004) and shoots (Yamamoto and Kiss, 2002). Studies also indicate that disrupting F-actins with Lat-B enhances the movement of some amyloplasts (Hou et al., 2004), but reduces the movement of the vast majority of amyloplasts (Palmieri and Kiss, 2005; Saito et al., 2005). Instead, inhibition of myosin ATPase activity with BDM increases amyloplast movement in vertically oriented hypocotyls. After reorientation, amyloplasts exhibit less displacement in response to gravity, continuing to saltate, and the organ curves less in response to gravity (Palmieri et al., 2007).

In summary, the classical model of static sensing of amyloplasts sedimenting to the lowest point in the sensing cell does not generally comply with the following observations: (i) the sedimentation speed of amyloplasts is orders of magnitude slower than the speed required to explain recorded presentation times, (ii) highly sensitive gravitropic response and signal integration (iii) gravity can also be sensed via much weaker sensors than amyloplasts (although with greater variance of response curves), (iv) applying mechanical or thermal noise results in enhanced sensing, (v) saltatory movements of statoliths. Moreover, the role of the cytoskeleton in gravitropism, both at the level of signal perception and transduction, needs to be understood.

## 4. Current alternative models and directions

We now present some current alternative models and directions that attempt to incorporate and interpret these experimental observations. We focus on the role of the cytoskeleton in both signal-to-noise modulation and signal transduction, and the dynamic sensing model which suggests stochastic events enhance gravisensing via different approaches. The underlying notion is that noise has a central role in regulating and enhancing the gravitopic repones in plants.

### 4.1. The role of the cytoskeleton in signal-to-noise modulation

Several studies have proposed that amyloplasts and the actomyosin system are part of a feedback mechanism that fine-tunes the gravitropic response (Hou et al., 2004; Palmieri and Kiss, 2005; Saito et al., 2005). Indeed the cytoskeleton has been found to affect saltatory movements of amyloplasts, as mentioned in Section 3. One can interpret these results also in terms of changes in the signal-to-noise ratio (Palmieri et al., 2007): As mentioned earlier, the movement of the vast majority of amyloplasts is inhibited when the actin cytoskeleton is disrupted (including saltatory movements, i.e., reduction of noise). The few mobile amyloplasts therefore impart a gravity signal, which, in the absence of noise, may be perceived as amplified. Therefore, amyloplasts may be seen as indicators of noise and the direction of gravity signal. In this context the cytoskeleton can be seen as modulating the signal-to-noise ratio.

### 4.2. Dynamic sensing: stochastic events enhance gravisensing

Hasenstein (2009) attempts to propose an alternative model to the classic picture of amyloplast sedimentation. Following the caveats presented in Section 3, the gravisensing system must respond to mechanical and thermodynamic noise, be compatible with the undisputed function of statoliths but must also be able to function in the absence of amyloplasts. Rather than perceiving gravity statically at the lowermost site of sedimentation, which produces one stimulus per settled statolith, it is suggested (Hasenstein, 2009) that stochastic events due to thermal and mechanical noise (for example due to cytoplasmic streaming (Kato et al., 2002; Morita et al., 2002) and cellular activities such as cytoskeletal tread milling), affect any susceptible particle in two ways: (i) effective lowering of mechano-stimulation, allowing smaller masses to act as statoliths, and (ii) gravisensing occurs via continuous small-distance sedimentation, allowing the amplification, and integration of the signal.

Let us expand on these two points. Stochastic events cause smaller masses to exceed the minimum energy needed to activate the ensuing signaling process. This is in agreement with *stochastic resonance*, a stochastic process where noise effectively lowers the threshold of mechano-stimulation, thus allowing to discern weak signals (Wiesenfeld and Moss, 1995). Stochastic resonance has been found to play a role in many mechano-transduction systems, ranging from crayfish (Douglass et al., 1993a) to hair cells in animal and human auditory system (Jaramillo and Wiesenfeld, 1998, 2000; Indresano et al., 2003). Secondly, it is suggested (Hasenstein, 2009) that gravisensing occurs via short distance sedimentation, rather than static perception at the lowermost site of sedimentation which produces one stimulus per settled statolith. This is in accordance with the saltatory motion described in Section 3. In this case sedimentation events take place continuously, constantly stimulating the system, allowing the amplification, and integration of the signal. The resulting signal is therefore continuously updated and dynamic.

In this context, heavier particles may lead to more interactions (and more energetic ones) between statoliths and membranes, cytoskeletal elements, or other sensitive structures, including vacuoles (Kato et al., 2002). Gravity affects the distribution of the combined statoliths and detectable events, which increase in the direction of the gravity vector.

### 4.3. The role of the cytoskeleton in signal transduction

The interaction between amyloplasts and F-actin has already been mentioned earlier in relation to gravity perception and noise. In the context of signal transduction, it has been proposed that the sedimentation of amyloplasts through the F-actin network inside the statocyte may possibly impact a mechano-sensitive surface at the cell periphery (for reviews, see Perbal and Driss-Ecole, 2003). Alternatively, amyloplasts may disrupt the tensional integrity of the F-actin network, mechanically stimulating ion channels in the plasma membrane Palmieri et al., 2007.

Hasenstein (2009) also suggests that some correcting activity is associated with the (F-actin) cytoskeleton, i.e., regulation of the signal transduction: Short-distance sedimentation of statoliths and other particle lead to interactions between statoliths and membranes, cytoskeletal elements, or other sensitive structures, including vacuoles (Kato et al., 2002). These events may trigger a signal associated with the membrane, such as the opening or activation of stretch-activated channels (Perbal et al., 2004) or auxin efflux carriers (PIN3 proteins). Related investigations on mechano-transduction in human leukemia cells show that F-actin disassembly reduces the amplitude of stretch-activated currents but not the probability of channel opening (Staruschenko et al., 2005), suggesting that some correcting activity associated with the (F-actin) cytoskeleton is necessary to regulate the channels. As long as gravistimulation occurs, basal channels either on the endoplasmic reticulum or plasma membrane may open and provide discrete but frequent signals that indicate the direction of the gravitational vector. Disruption of the actin filaments may prevent closure and cause persistent channel conductivity, compatible with experimental observations where disrupting F-actin enhances the sensitivity and cause an over-response in curvature for roots (Hou et al., 2003, 2004) and shoots (Yamamoto and Kiss, 2002).

## 5. Discussion

In this short review we focused on the role of noise in the gravitropic response of plants. We described the conventional model based on the sedimentation of high-mass statoliths to the lowest part of the sensing cell, and continued to present experimental observations that cannot currently be understood within this framework. We then pointed out some current alternative models and directions, that attempt to incorporate and interpret these experimental observations. These include the role of the cytoskeleton in signal-to-noise modulation and signal transduction, and the dynamic sensing model, where stochastic events enhance gravisensing. The common thread to all of these is the idea that intrinsic noise has a central role in regulating and enhancing the gravitopic repones in plants. Having said that, much is still not fully understood in the sensing and signal transduction mechanisms, and more so the role of noise in these. We propose here a number of directions that seem to have either not been explored, or that are still poorly understood.

- Accommodation – gravitropic sensitivity is found to depend on the strength of the stimulus (more sensitive for weaker stimuli), reminiscent of the Weber-Fechner law of perception (Moulia et al., 2006; Moulia and Fournier, 2009). Indeed, root statocytes have been shown to be more sensitive in microgravity compared to those grown on a 1 g centrifuge in space (Perbal and Driss-Ecole, 2003).
- Teasing apart sources of noise at different stages of perception: the initial sensing, signal transduction, and finally organ response. An example in gravitropism is that any external mechanical stimulus can only affect the initial sensory system, not the down-stream biochemical signal transduction. This sort of argument is more elusive when differing between the signal transduction and the organ response.
- Mathematical modeling – in recent years there has been a rise in mathematical modeling at the molecular, cellular and organ level, and has proven to offer valuable insights in the tropism mechanisms (e.g., Shafrir and Forgacs, 2002; Moulia and Fournier, 2009; Bastien et al., 2013 and many others). Current mathematical models lack a stochastic framework that would address the addition of noise to the system. One way of taking this into consideration is to add noise into dynamic equations of, e.g., the curvature of the shoot (Bastien et al., 2013), reminiscent of a Langevin equation. In line with the previous point, it is important to distinguish between different sources of noise, and thus incorporate them in a meaningful way. By doing so one may attribute the noise to different terms: the response, proprioception, graviception or even the stimulus itself. Secondly, the stochastic mechanisms mentioned here require modeling and may be incorporated within an adequate integrative mathematical framework. Such an understanding may help pose a new understanding of the problem.

### Conflict of interest statement

The Guest Associate Editor Karen Alim declares that, despite being affiliated to the same institution as the authors, the review process was handled objectively and no conflict of interest exists. The authors declare that the research was conducted in the absence of any commercial or financial relationships that could be construed as a potential conflict of interest.
